# Phase III randomised controlled trial on PSMA PET/CT guided hypofractionated salvage prostate bed radiotherapy of biochemical failure after radical prostatectomy for prostate cancer (PERYTON-trial): study protocol

**DOI:** 10.1186/s12885-022-09493-5

**Published:** 2022-04-15

**Authors:** F. H. E. Staal, J. Janssen, C. L. Brouwer, J. A. Langendijk, K. Ng Wei Siang, E. Schuit, I. J. de Jong, J. F. Verzijlbergen, R. J. Smeenk, S. Aluwini

**Affiliations:** 1 Department of Radiation Oncology, University Medical Centre Groningen, Hanzeplein 1, Postbus 30 001, 9700 RB Groningen, The Netherlands; 2 Julius Centre for Health Sciences and Primary Care, University Medical Centre Utrecht, Utrecht University, Utrecht, The Netherlands; 3 Department of Urology, University Medical Centre Groningen, Groningen, The Netherlands; 4 Department of Nuclear Medicine, Radboud University Nijmegen Medical Centre, Nijmegen, The Netherlands; 5 Department of Radiation Oncology, Radboud University Nijmegen Medical Centre, Nijmegen, The Netherlands

**Keywords:** Salvage radiotherapy, Biochemical recurrence, Hypofractionation, PSMA PET/CT scan, Prostate cancer, Prostatectomy

## Abstract

**Background:**

Salvage external beam radiotherapy (sEBRT) for patients with a biochemical recurrence (BCR) after radical prostatectomy provides a 5-year biochemical progression-free survival up to 60%. Multiple studies have shown that dose escalation to the primary prostate tumour improves treatment outcome. However, data is lacking on the role of dose escalation in the recurrent salvage setting. The main objective of the PERYTON-trial is to investigate whether treatment outcome of sEBRT for patients with a BCR after prostatectomy can be improved by increasing the biological effective radiation dose using hypofractionation. Moreover, patients will be staged using the PSMA PET/CT scan, which is superior to conventional imaging modalities in detecting oligometastases.

**Methods:**

The PERYTON-study is a prospective multicentre open phase III randomised controlled trial. We aim to include 538 participants (269 participants per treatment arm) with a BCR after prostatectomy, a PSA-value of < 1.0 ng/mL and a recent negative PSMA PET/CT scan. Participants will be randomised in a 1:1 ratio between the conventional fractionated treatment arm (35 × 2 Gy) and the experimental hypofractionated treatment arm (20 × 3 Gy). The primary endpoint is the 5-year progression-free survival after treatment. The secondary endpoints include toxicity, quality of life and disease specific survival.

**Discussion:**

Firstly, the high rate of BCR after sEBRT may be due to the presence of oligometastases, for which local sEBRT is inappropriate. With the use of the PSMA PET/CT before sEBRT, patients with oligometastases will be excluded from intensive local treatment to avoid unnecessary toxicity. Secondly, the currently applied radiation dose for sEBRT may be too low to achieve adequate local control, which may offer opportunity to enhance treatment outcome of sEBRT by increasing the biologically effective radiotherapy dose to the prostate bed.

**Trial registration:**

This study is registered at ClinicalTrials.gov (Identifier: NCT04642027). Registered on 24 November 2020 – Retrospectively registered. The study protocol was approved by the accredited Medical Ethical Committee (METc) of all participating hospitals (date METc review: 23-06-2020, METc registration number: 202000239). Written informed consent will be obtained from all participants.

## Highlights

• This is the first PSMA PET/CT based study addressing the role of hypofractionation in salvage external beam radiation therapy (sEBRT);

• Participants will be randomised 1:1 to conventional fractionated sEBRT (35 × 2 Gy) or hypofractionated sEBRT (20 × 3 Gy);

• The PSMA PET/CT-scan will be used for staging of all participants at baseline;

• The primary endpoint is 5-year progression-free survival after treatment;

• All participants will receive early sEBRT (PSA < 1.0 ng/mL) without the use of androgen deprivation therapy;

• A quality assurance program was undertaken, to ensure protocol compliance within the PERYTON-trial;

• An interim analysis will be performed after the inclusion of 25% (*n* = 135) of the total number of required participants, to identify any early signs of excessive (unexpected) acute toxicity.

## Background

According to the Dutch Cancer Registry, prostate cancer (PCa) is the first most diagnosed cancer in men with 12.800 new cases and 2.954 deaths in 2018 in the Netherlands [1]. Radical prostatectomy (RP) as primary treatment provides excellent control in localized PCa. After RP, however, approximately 20-40% of patients will develop a biochemical recurrence (BCR) within 5 years. The site of relapse is predominately local (35 to 54%) [2–4]. The only curative treatment option for BCR after RP is salvage external beam radiation therapy (sEBRT). However, 40-50% of the patients treated with sEBRT develop a secondary BCR [5]. This relatively high percentage of a secondary BCR after sEBRT necessitates the need to improve the treatment outcome of sEBRT and subsequently to reduce the need for more aggressive palliative treatment strategies.

Since the introduction of the prostate-specific membrane antigen (PSMA) positron emission tomography (PET)/computed tomography (CT) scan, the detection of oligometastases at very low prostate specific antigen (PSA) levels has significantly improved [6]. PSMA PET/CT can detect nodal metastases as small as 2.4 mm with up to 80% sensitivity and 97% specificity [7]. Recent studies have demonstrated significant changes (30 to 76%) in management decisions for recurrent PCa by incorporating PSMA-PET/CT in an early stage of BCR after RP [6, 7].

Firstly, the high rate of BCR after sEBRT may be due to the presence of oligometastases, for which local sEBRT is inappropriate. With the use of the PSMA PET/CT before commencing sEBRT, patients with oligometastases will be excluded from intensive local treatment to avoid unnecessary toxicity [7–10]. Furthermore, the absence of active disease on PSMA PET/CT scans is an independent prognostic factor for high response to sEBRT [11]. These findings support applying sEBRT in patients with BCR without evidence of distant metastasis on PSMA-PET/CT scan in whom local recurrence in the prostate bed cannot be detected.

Secondly, the currently applied conventional radiation schedule for local sEBRT may be insufficient. This may offer opportunities to improve biochemical progression-free survival (bPFS) by increasing the biologically effective radiotherapy dose to the prostate bed using hypofractionation. Studies have shown that the α/β-ratio for PCa cells is 1.5 Gy, which is lower than that of surrounding normal tissues [12]. This means that PCa cells are more sensitive to fraction dose than surrounding tissues, which is the rationale for increasing the dose-per-fraction (hypofractionation) instead of the total dose. In doing so, the biological dose to the tumour cells, but not to the surrounding normal tissues (bladder and rectum), is expected to increase. Assuming an α/β of 4 Gy for bladder and rectum, the equivalent dose at 2 Gy (EQD2) of the hypofractionated schedule of 20 × 3 Gy is 70 Gy, which is equal to the 70 Gy in the standard arm [12].

In primary PCa, multiple randomised controlled trials (RCT) have shown non-inferiority of a 20 × 3.0 Gy = 60 Gy schedule over 39 × 2.0 Gy = 78 Gy, confirming its biological equivalence of both schedules regarding oncologic outcome and toxicity [13, 14]. Based on these results, we expect the experimental study regimen (20 × 3 Gy) will be biologically equivalent to 78 Gy: 8 Gy higher than the current standard of 70 Gy standard. Although hypofractionation is standard of care in the primary PCa setting, data on hypofractionated sEBRT are scarce. A recently published systematic review supports the tolerance of hypofractionated sEBRT, but stresses the importance of conducting RCTs comparing hypofractionated with standard fractionated sEBRT [15].

To investigate the potential benefit of hypofractionated sEBRT, we will start a phase III RCT (the PERYTON-trial, ClinicalTrials.gov Identifier: NCT04642027). The aim of the PERYTON-trial is to assess whether a dose escalated hypofractionated regimen will improve the 5-year progression-free survival of sEBRT selected with PSMA PET/CT. Furthermore, we will assess the influence of hypofractionation on toxicity, quality of life (QoL) and survival.

## Methods/design

### Study design

**Figure 1:**
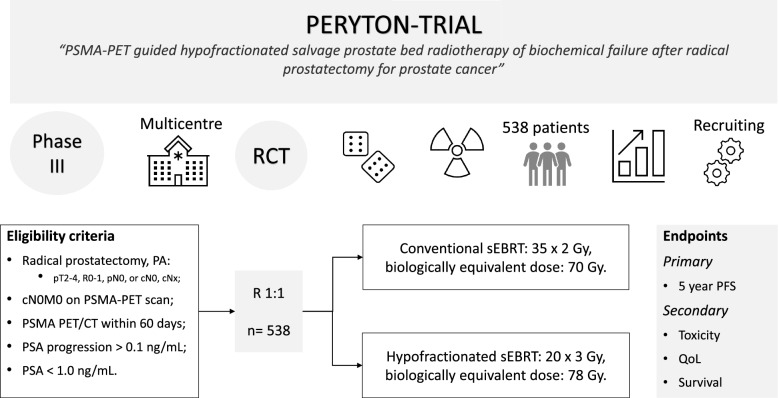
**Fig. 1** The design of the PERYTON-trial

The PERYTON-trial is a prospective multicentre phase III randomised controlled trial. (Fig. 1).

Participating centres are radiotherapy treatment centres in the Netherlands (updated list available on ClinicalTrials.gov).

All eligible participants will be randomly assigned (1:1) to receive:

1. Current standard: conventional sEBRT, 35 × 2 Gy, biologically equivalent dose: 70 Gy.
2. Experimental arm: hypofractionated sEBRT, 20 × 3 Gy, biologically equivalent dose: 78 Gy.

Participants will be stratified by PSA level (≤0.2 ng/mL vs > 0.2- ≤0.5 ng/mL vs > 0.5- < 1.0 ng/mL) and local recurrence on the PSMA PET/CT (presence versus absence).

### Participants

All patients with a BCR, with a PSA value < 1.0 ng/mL, after radical prostatectomy for PCa and staged using a PSMA PET/CT scan (without any evidence of lymph nodes or distant metastases) are eligible for inclusion. Detailed in-and exclusion criteria are summarized in Table 1.

**Table 1:** Inclusion and exclusion criteria of the PERYTON-trial. Patients are eligible if they meet all criteria

Inclusion criteria	Exclusion criteria
• Patients with prostate adenocarcinoma treated with radical prostatectomy; • Tumour stage pT2-4, R0-1, pN0, or cN0, cNx according to the UICC TNM 2009, only with Gleason score available; • A recent PSMA-PET scan (< 60 days) without evidence of lymph nodes or distant metastases; • PSA progression after prostatectomy defined as two consecutive rises with the final PSA > 0.1 ng/mL or 3 consecutive rises. The first value must be measured ≥6 weeks after radical prostatectomy; • PSA at inclusion < 1.0 ng/mL; • WHO performance status 0–2 at inclusion; • Age at inclusion between 18 and 80 years; • Written (signed and dated) informed consent prior to registration.	• Prior pelvic irradiation, (chemo) hormonal therapy or orchiectomy; • Previous or concurrent active invasive cancers, other than superficial non-melanoma skin cancers; • Patients with positive lymph nodes or distant metastases based on the surgical specimen of lymphadenectomy or the following minimum diagnostic workup: PSMA-PET/CT scan, 60 days prior to registration; • Double-sided metallic hip prosthesis; • Inability or unwillingness to understand the information on trial-related topics, to give informed consent or to fill out QoL questionnaires.

### Primary endpoint

The primary endpoint is 5-year progression-free survival (PFS), defined as biochemical progression, clinical progression, loco-regional progression, distant progression or the start with hormonal therapy, whichever occurs first. Biochemical progression will be defined as a PSA value of 0.2 ng/mL higher than the PSA Nadir (defined as the lowest PSA value ≥3 months after completion of treatment). Participants with no change or an increase in PSA level after sEBRT will be defined as non-responders.

### Secondary endpoints

- Acute grade ≥ 2 gastrointestinal (GI) and genitourinary (GU) toxicities measured 3 months after treatment using both physician-reported score (Common Terminology Criteria for Adverse Events (CTCAE), version 5.0) and patient-reported questionnaires (RTOG Acute Radiation Morbidity Criteria using adapted RTOG/EORTC acute Radiation Morbidity questionnaires);
- 3- and 5-year late grade ≥ 2 GI and GU toxicities, measured using both physician-reported score (CTCAE version 5.0 criteria) and patient-reported questionnaires (RTOG/EORTC criteria);
- QoL, using the European Organization for Research and Treatment of Cancer (EORTC) QoL thirty item score questionnaires (QLQ-C30) for health-related QoL and the EORTC QoL prostate carcinoma module (QLQ-PR25) is used;
- Metastasis-free survival: Randomisation date to the date of metastases reported by imaging;
- PCa-specific mortality;
- Overall survival at 5 years from date of randomisation.

### Time schedule

The PERYTON-trial started with patient accrual in September 2020. The follow-up starts from the last treatment day, and the main analysis addressing the primary endpoint (5-year PFS) is planned after 5 years of follow-up (i.e., 5 years since the inclusion of the last participant in the study).

### Radiotherapy

#### Radiation dose in sEBRT

A dose–effect relationship has been reported in sEBRT, indicating a 2.0% improvement in bPFS for each additional Gy [16]. One recent RCT, the SAKK-trial 09/10, compared conventional dose schedule (32 × 2 Gy = 64 Gy) to a dose-escalated schedule (35 × 2 Gy = 70 Gy) and showed that dose-intensified sEBRT was not superior to conventional-dose sEBRT [17]. However, this escalated dose of only 4 Gy in the SAKK-trial is probably too low to improve outcome. Studies on dose escalation in primary PCa radiotherapy reported higher GI toxicity, while hypofractionation is proven effective with comparable acute and late toxicity results [13, 14]. This positive experience with hypofractionated radiotherapy in primary PCa may give the opportunity to apply hypofractionation as a dose escalation technique in sEBRT. Data on hypofractionated sEBRT are scarce with only few non-randomised reports. These reports showed adequate disease control and acceptable rates of acute and late side effects, indicating the need to investigate the role of hypofractionation in sEBRT in an RCT [18–21].

#### Quality assurance

A pre-trial quality assurance (QA) process was undertaken to ensure validity and reliability of radiation therapy treatment data to improve protocol compliance within all including centres within this trial. Completion of the pre-trial QA program is mandatory before a centre can start with accrual. At this moment, 6 centres completed the pre-trial QA program. Analyses of the pre-trial QA showed that the dose constraints of the PERYTON-trial are feasible [22].

#### Preparations and positioning

It is recommended to scan and treat patients with a comfortably full bladder. The use of an endorectal balloon is allowed. Local daily online position verification and correction protocols may be applied, being identical in both treatment arms. The use of feet and knee support is recommended.

#### Delineation

Gross tumour volume (GTV) is defined, if present, as the visible local lesion on planning CT (matched with the positive PSMA PET/CT scan). Clinical target volume (CTV) will be defined by any of the published consensus guidelines such as EORTC [23], RTOG [24] or Wiltshire et al. [25] The planning target volume (PTV) margin is according to the local verification protocol. The organs at risk (OAR) include the bladder, rectum, bilateral femora, penile bulb and the large and small bowel.

#### Planning

Highly conformal treatment techniques are used, i.e., intensity modulated radiation therapy (IMRT) or volumetric modulated arc therapy (VMAT). The prescribed dose to the prostate bed is:

- Arm 1: 35 × 2.0 Gy = 70 Gy.
- Arm 2: 20 × 3.0 Gy = 60 Gy.

If a local recurrence is detected within the prostatic bed on PSMA PET/CT-scan, it is recommended to deliver a boost to the GTV. This boost should consist of:

- Arm 1: An equivalent dose of 74 – 78 Gy (concomitant or sequential);
- Arm 2: An equivalent dose of 62 – 63 Gy (concomitant or sequential).

#### Target coverage

At least 99% of the PTV should receive 95% of the prescribed dose: PTV V95% ≥ 99%. Variation of 4% in the V95% (95-99%) is acceptable if necessary to keep the OAR dose within the protocol constraints. Furthermore, 100% of the CTV should receive 95% of the prescribed dose: CTV V95% = 100%.

### Organ at risk constraints

All OAR constraints are summarized in Table 2. A variation of ≤5% of rectal and bladder volumes exceeding the protocol dose constraints are accepted, if needed to reach an acceptable target coverage. The acceptance of rectal volumes beyond this additional 5% of the constraints will be considered a major protocol violation.

**Table 2:** Overview of dose constraints to the organs at risk per treatment arm

Organ	Constraints	Arm 1 (35x2Gy)	Arm 2 (20x3Gy)
Rectum	V^a^70	< 5%	
	V65	< 15%	
	V60		< 5%
	V55		< 15%
Rectal wall	V25		< 80%
	V30	< 80%	
	V35		< 50%
	V41	< 50%	
	V50		< 25%
	V60	< 25%	
	Mean Dose	< 30Gy	< 25Gy
Anus	Mean Dose	< 25Gy	<22Gy
Anal wall	V25		< 30%
	V30	< 30%	
Bladder	Dose_0.1cc_	< 105%	< 105%
	V70	< 25%	
	V60	< 50%	< 25%
	V50		< 50%
Femoral head	V45		< 10%
	V55	< 10%	
Penile bulb		ALARA^b^	ALARA

^a^Volume; ^b^As low as reasonably achievable

### Systemic treatment: no androgen deprivation therapy within the PERYTON-study

Two phase III RCTs have shown superior outcomes after postoperative sEBRT when androgen deprivation therapy (ADT) was added (6 months of LHRH-analogues or 24 months of bicalutamide) [26, 27]. However, both trials used low dose sEBRT (64.8 – 66 Gy) and included patients with high PSA levels (2.0 to 4.0 ng/mL). Therefore, the reported benefit of ADT was probably at least partly compensating for low dose sEBRT. Additionally, the benefit was limited to those patients with a high PSA level, so at higher risk for metastases, rather than an additive local benefit. This hypothesis is supported by the use of conventional imaging modalities in both studies to exclude metastases, which could enhance the benefit of ADT in both trials. Post hoc analysis by Shipley et al. showed no benefit of ADT for patients with a PSA level < 0.7 ng/mL [27]. This PSA level will apply to the majority of our population. Using the PSMA PET/CT in an early salvage setting, at low PSA levels of ≤1.0 ng/mL, it is very likely that the use of ADT in the PERYTON-trial will have no added value. In conclusion, the use of ADT is not allowed within the PERYTON-trial.

### Follow-up evaluation

Participants will be followed according to the time schedule in Table 3.

**Table 3:** Summary of the follow-up in the first 5 years after RT

Required investigation	Inclusion (within 4 wk. prior to randomisation)	During treatment		Follow-up							
		4 wks	7 wks	1.5 m after RT	3 m after RT	6 m after RT	9 m after RT	12 m after RT	18 m after RT	24 m after RT	Every 12 m (up to 60 m)
Eligibility check	X										
Informed consent	X										
Characteristics	X										
Medical history / medication list	X			X	X	X	X	X	X	X	X
WHO performance status	X			X		X		X	X	X	X
pT-stage and GSS	X										
PSA	X			X	X	X	X	X	X	X	X
Testosterone	X							X		X	X
PSMA/PET CT	X										
Radiotherapy treatment planning		X									
Survival / progression status				X	X	X	X	X	X	X	X
Surveys											
CTCAE v5	X	X^a^		X	X	X	X	X	X	X	X
RTOG-EORTC Toxicity	X	X^a^		X	X	X	X	X	X	X	X
EORTC-QLQ-PR25	X				X	X	X	X	X	X	X
EORTC-QLQ-C30	X					X	X	X	X	X	X
SAE				X	X	X	X	X	X	X	X

^a^Have to be completed at the end of radiotherapy (4 weeks in the experimental arm and 7 weeks in the standard arm)

### Sample size considerations

A systematic review and meta-analysis reported that each additional Gy in the conventional sEBRT-arm is possibly associated with a 2% higher bPFS, independent of other factors. These results imply that our biological dose escalated effect with hypofractionation will result in 16% higher bPFS (8 Gy × 2% per Gy). However, this meta-analysis did not include series using a radiotherapy dose above 70 Gy. In higher dose series as proposed in this study, the relative benefit may be lower and consequently an expected benefit of 16% may be too optimistic. Therefore, a conservative 12% higher 5-year bPFS in the experimental arm of 20 fractions of 3 Gy compared to the conventional sEBRT schedule of 35 fractions of 2 Gy (70 Gy) has been chosen. This estimated benefit of 12% is also supported by a meta-analysis including conventional and hypofractionated regimens addressing the influence of fraction size and the total dose effect on bPFS and toxicity [28].

The results of the GETUG-AFU 16 trial showed a 5-year bPFS of 62% with the conventional fractionated radiotherapy protocol [26]. We used this percentage (62%) for the 5-year bPFS in our conventional sEBRT treatment arm. Following the above mentioned literature, we hypothesize that with the experimental regimen the bPFS can be improved from 62 to 74%. Using an alpha of 5% and a study power of 81%, the required sample size is 235 participants per treatment arm. Assuming a dropout of 12%, we need to recruit 269 participants per treatment arm: 538 in total.

### Statistical analysis

The incidence of progression-free survival will be compared between the two treatment arms using Cox proportional hazards regression analysis to calculate hazard ratios and 95% confidence intervals. Acute and late toxicity rates will be summarized in terms of the proportion of participants in each arm. Log-binominal regression will be used to compare the individual highest grade of acute toxicity and the individual highest grade of late toxicity between both treatment arms. Relative risks will be calculated with 95% confidence intervals. For the analysis of QoL data, a comparison of the change in QoL from baseline level and at different time points will be performed between both treatment arms. The statistical analysis of QoL will be performed by a random effects linear regression model. Stratified randomisation will be taken into account in all analyses by adding the stratification variables to the respective regression models.

In sensitivity analyses, we will repeat all analyses, but with adjustment for potentially prognostic factors (age, pre-operative tumour stage, PSA level, time between radical prostatectomy and biochemical relapse, and comorbidity) or confounding factors (treatment centre).

### Interim analysis

Dose-limiting constraints for rectum and bladder will be used (Table 2) and a QA process is mandatory before the start of inclusion in the participating centres. Toxicity is expected to be equivalent in both arms. Nevertheless, a slight increase in acute toxicity in the experimental arm might be expected due to the short overall treatment time [29]. Therefore, we will perform an interim analysis after the inclusion of 25% of the estimated number of participants (135 participants) to identify any early signs of excessive (unexpected) acute toxicity. Grade 3 CTCAE acute toxicity will be used for this interim analysis; i.e., > 5% difference in grade 3 toxicity between the experimental and control arm. If this acute toxicity difference exceeds 5%, a formal interim analysis will be performed with a nominal *p* value < 0.001 according to the Haybittle-Peto alpha spending function, to indicate statistical significance [30]. The interim analysis will be performed by an independent statistician, and the results will be shared with the principal investigators who will judge after informing the ethical committee whether continuation of the study is safe.

## Discussion

Salvage external beam radiation therapy (sEBRT) is an established treatment option for BCR after RP, with a 5-year bPFS up to 60%. Which means that 40% of the patients receiving sEBRT experience a second BCR after radiotherapy. This suggests that sEBRT does not benefit those patients with a second BCR, while exposing them to treatment-related side effects. Hence, there is a need to improve the treatment outcome of sEBRT.

Predisposing factors for BCR after sEBRT may either originate at the primary tumour site, indicating insufficient local treatment, or from disease outside the prostate bed (i.e., occult metastases), suggesting metastatic disease for which local sEBRT is not appropriate.

The PERYTON-study aims to increase the effectiveness of sEBRT after radical prostatectomy by:

1. Excluding patients with metastatic disease for sEBRT with the (early) use of the PSMA-PET/CT scan as screening imaging modality before the inclusion of patients.
2. Using an hypofractionated radiotherapy schedule of 20 × 3 Gy in sEBRT to escalate the biological effective radiation dose to the prostate bed.

In conclusion, the PERYTON-trial is, to our knowledge, the first prospective phase III RCT, using the PSMA PET/CT-scan as standard imaging modality and investigating the potential benefit of a hypofractionated sEBRT schedule of 20 × 3 Gy = 60 Gy compared to the conventional fractionated standard of 35 × 2 Gy = 70 Gy.

## Data Availability

Not applicable.

## Abbreviations

PCa: Prostate cancer
RP: Radical prostatectomy
BCR: Biochemical recurrence
sEBRT: Salvage external beam radiotherapy
PSMA PET/CT: Prostate-specific membrane antigen positron emission tomography/computed tomography
bPFS: Biochemical progression-free survival
EQ D2: Equivalent Dose at 2 Gy
QoL: Quality of life
RCT: Randomised controlled trial
PFS: Progression-free survival
PSA: Prostate specific antigen
GI: Gastrointestinal
GU: Genitourinary
QA: Quality assurance
GTV: Gross tumour volume
CT-scan: Computed tomography scan
CTV: Clinical target volume
PTV: Planning target volume
IMRT: Intensity modulated radiation therapy
VMAT: Volumetric modulated arc therapy
OAR: Organ at risk
ADT: Androgen deprivation therapy
